# Estradiol-17β Regulates Expression of Luteal DNA Methyltransferases and Genes Involved in the Porcine Corpus Luteum Function In Vivo

**DOI:** 10.3390/ijms22073655

**Published:** 2021-04-01

**Authors:** Piotr Kaczynski, Monika Baryla, Ewelina Goryszewska, Agnieszka Waclawik

**Affiliations:** Institute of Animal Reproduction and Food Research, Polish Academy of Sciences, Tuwima 10, 10-748 Olsztyn, Poland; m.baryla@pan.olsztyn.pl (M.B.); e.goryszewska@pan.olsztyn.pl (E.G.)

**Keywords:** corpus luteum, estradiol-17β, DNA methyltransferases, pig

## Abstract

The corpus luteum (CL) is a temporary endocrine gland vital for pregnancy establishment and maintenance. Estradiol-17β (E2) is the major embryonic signal in pigs supporting the CL’s function. The mechanisms of the luteoprotective action of E2 are still unclear. The present study aimed to determine the effect of E2 on luteal expression of factors involved in CL function. An in vivo model of intrauterine E2 infusions was applied. Gilts on day 12 of pregnancy and the estrous cycle were used as referential groups. Concentrations of E2 and progesterone were elevated in CLs of gilts receiving E2 infusions, compared to placebo-treated gilts. Estradiol-17β stimulated luteal expression of DNA-methyltransferase 1 (DNMT1), but decreased expression of DNMT3B gene and protein, as well as DNMT3A protein. Similar results for DNMT3A and 3B were observed in CLs on day 12 of pregnancy compared to day 12 of the estrous cycle. Intrauterine infusions of E2 altered luteal expression of the genes involved in CL function: *PTGFR*, *PTGES*, *STAR*, *HSD17B1*, *CYP19A1,* and *PGRMC1*. Our findings indicate a role for E2 in expression regulation of factors related to CL function and a novel potential for E2 to regulate DNA methylation as putative physiological mechanisms controlling luteal gene expression.

## 1. Introduction

Early pregnancy in mammals, including pigs, is a critical period that is dependent on molecular interactions between developing embryos and the maternal organism. At this stage, there is limited time during which the uterine luminal epithelium is favorable to blastocyst implantation, which has been defined as uterine receptivity [1]. The key processes determining the establishment and development of pregnancy are the maternal recognition of pregnancy and implantation. Maternal recognition of pregnancy in the pig occurs between days 10 and 13 after fertilization [2] and during this period porcine conceptuses by secreting a plethora of factors to signal their presence in the reproductive tract (reviewed in [3]). Estrogens are the primary embryonic signals in pigs and are secreted in a biphasic manner on days 11 and 12 of the pregnancy and on days 15–30 of the pregnancy [4]. The corpus luteum (CL) is a transient endocrine organ, and its main function is the synthesis and secretion of progesterone (P4) which, in turn, primes uterine receptivity, enabling conceptus development and implantation [5,6]. In pigs, proper CL function during pregnancy is important, as it is the major source of progesterone throughout the whole gestation [7].

Increased abundance of estradiol-17β (E2) in the uterine lumen during early pregnancy has been found to be related to its luteoprotective action. Administration of exogenous E2 results in the maintenance of high levels of luteal LH receptor expression [8]. It has been shown in pigs that the direct action of E2 on CL stimulates progesterone secretion [9]. Acting on the endometrium, E2 induces mechanisms altering the secretion of prostaglandin F2α (PGF2α) in an endocrine (into utero-ovarian venous drainage) to exocrine (into uterine lumen) manner, which protects the corpora lutea from the luteolytic action of PGF2α [2]. Moreover, E2 regulates prostaglandin (PGE2 and PGF2α) synthesis and signaling in the endometrium [10,11,12]. It favors endometrial synthesis of PGE2, which is transported to the ovaries to exert a luteoprotective effect [10,13,14]. The expression of the estrogen receptor 1 (*ESR1*) gene in the porcine corpus luteum is upregulated on day 12 of the pregnancy when compared to earlier stages of pregnancy (days 8 and 10) [15]. The expression profiles of genes related to processes involved in corpus luteum function have also been characterized. Genes such as progesterone receptor membrane component 1 (*PGRMC1*), steroidogenic acute regulatory protein (*STAR*), 3beta-hydroxysteroid dehydrogenase/delta(5)-delta(4)isomerase (*HSD3B1*), nuclear receptor subfamily 5 group A member 1 (*NR5A1*), prostaglandin F2α receptor (*PTGFR*), prostaglandin E2 receptor 4 (*PTGER4*), and vascular endothelial growth factor (*VEGFA*) have been found to be differentially expressed in the CL during the time corresponding to maternal recognition of pregnancy in pigs [13,15]. Interestingly, the luteal concentration of E2 also increases on day 12 of pregnancy [15].

Though the luteoprotective effect of porcine embryonic signal (E2) has been indicated, its role in mechanisms underlying the physiological functioning of the CL is still far from known. Intriguingly, studies on tumor growth have revealed the great potential of E2 in the induction of changes related to DNA methylation involving DNA methyltransferases (DNMT1, DNMT3A, and DNMT3B) [16,17]. E2-induced epigenetic processes have been linked to mechanisms responsible for tumorigenesis and metastasis (reviewed in [18]), including human adenocarcinomas [19]. Given that the dynamics of luteal tissue growth are similar to the growth of tumors it could be hypothesized that the E2-induced epigenetic mechanisms responsible for cancer development may be also involved in the physiological regulation of corpus luteum function. Interestingly, the expression of genes involved in ovarian function, such as cytochrome P450 family 1 subfamily A member 1 (aromatase; *CYP19A1)* [20,21], oxytocin [22] or inhibin α [23], has been shown to be altered due to DNA sequence methylation. However, the importance of epigenetic processes in regulating the physiological function of CL and the role of embryonic signals in these regulations have not been evidenced in any species.

Thus, in the present study, we aimed to determine whether primary porcine embryonic signal-estradiol-17β may affect the expression of genes and proteins regulating DNA methylation (DNMT1, DNMT3B, and DNMT3B), and genes involved in luteal function, in the pig in vivo. In the present study, we used an in vivo model, applying a novel approach of intrauterine infusions of E2, which enabled us to study the effects of E2 in concentrations close to the physiological levels secreted by embryos into the uterine lumen. Results from the in vivo model were compared with results from animals on day 12 of the estrous cycle and pregnancy (ex vivo approach), used as referential groups.

## 2. Results

### 2.1. The Effects on Intrauterine Estradiol-17β Administration In Vivo on Estradiol-17β and Progesterone Levels in CLs

**Figure 1:**
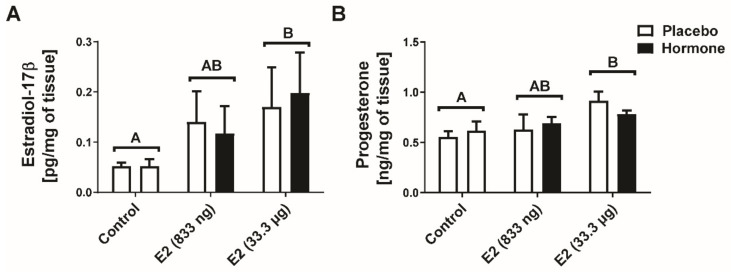
The concentration of estradiol-17β (E2) (**A**) and progesterone (**B**) in corpora lutea adjacent to placebo- and hormone-treated horns. Control—gilts receiving placebo infusions into both uterine horns. E2—gilts receiving infusions of either placebo into one randomly selected horn or E2 (833 ng or 33.3 µg/infusion) into contralateral horn. Data are expressed as the mean ± SEM. Main effect of treatment was detected on luteal concentration of E2 and progesterone (E2-treated groups vs. the control group; *p* < 0.05). There was no significant main effect of the site of hormone administration (i.e., the placebo-treated uterine horn vs. the hormone-treated uterine horn within the same animal) on luteal concentration of E2 and progesterone. Therefore, the different letters (above two bars for each group) indicate statistically significant differences between the control gilts and high-dose of E2-treated gilts (*p* < 0.05).

In the present study, we determined whether the intrauterine infusions of E2 may be transferred by a local circulation to the ovaries and whether it may affect the luteal concentration of progesterone. Hence, E2 and P4 concentrations have been evaluated in luteal tissue of CLs collected from ovaries adjacent to the E2-treated and placebo-treated uterine horns in experimental groups, as well as in luteal tissue of CLs collected from ovaries collected from the control group (placebo infusions to both uterine horns). Using Two-Way ANOVA only the effect of E2 treatment was detected on luteal concentration of E2 and progesterone (E2-treated groups vs. the control group, *p* < 0.05). However, there was no significant main effect of the site of hormone administrations (i.e., the placebo-treated uterine horn vs. the hormone-treated uterine horn within the same animal) on luteal concentration of E2 and progesterone. The results of the radioimmunoassay revealed a higher concentration of E2 in CLs collected from gilts receiving an intrauterine infusion of E2 (33.3 µg/infusion), regardless if CL was adjacent or contralateral to E2-infused uterine horn compared to the control group receiving an infusion of placebo into both uterine horns (*p* < 0.05; Figure 1A). Moreover, we found higher progesterone levels (*p* < 0.05) in CLs collected from E2-treated gilts (33.3 µg/infusion) into one uterine horn and placebo infusion into the contralateral uterine horn compared to levels in CLs collected from gilts assigned to the control group (placebo infusion into both uterine horns; Figure 1B). Administration of E2 (833 ng/infusion) had an intermediate effect on both E2 and P4 levels in CLs compared to gilts receiving a placebo infusion into both uterine horns and animals receiving an intrauterine infusion of a higher dose of E2 (33.3 µg/infusion).

### 2.2. Intrauterine Infusion of E2 Affects DNMT Gene and Protein Expression In Vivo

**Figure 2:**
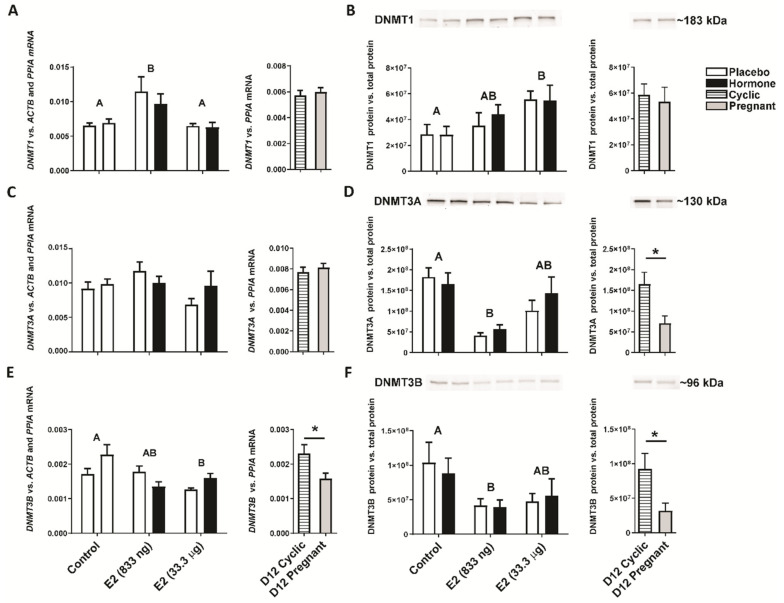
Luteal gene and protein expression of DNA methyltransferases (DNMT): DNMT1 (**A**,**B**); DNMT3A (**C**,**D**) and DNMT3B (**E**,**F**) in response to estradiol-17β (E2) treatment in vivo and in gilts on day 12 of the estrous cycle and pregnancy. Control—gilts receiving placebo infusions into both uterine horns. E2—gilts receiving infusions of either placebo into one randomly selected horn or E2 (833 ng or 33.3 µg/infusion) into the contralateral horn. As referential groups, gilts on day 12 of the estrous cycle (D12 Cyclic) and pregnancy (D12 Pregnant) were included. Values of *DNMTs* gene expression were normalized against geometrical mean of *ACTB* and *PPIA* expression values (luteal samples from in vivo model) or *PPIA* expression values (luteal samples from day 12 pregnant/cyclic pigs). Values of DNMTs protein expression were normalized against total protein content. Main effect of treatment was detected on luteal expression of DNMT1 mRNA and protein, DNMT3A protein, and DNMT3B mRNA and protein (E2-treated groups vs. the control group; *p* < 0.05). There was no significant effect of the site of hormone administration (i.e., the placebo-treated uterine horn vs. the hormone-treated uterine horn within the same animal) on luteal expression of DNMT1, DNMT3A and DNMT3B mRNA and protein. Data are expressed as the mean ± SEM. Different letters indicate statistically significant differences between the control- and hormone-treated gilts (*p* < 0.05). Asterisk indicates statistical differences between gilts on day 12 of the estrous cycle and pregnancy (*p* < 0.05).

Studying the expression of genes coding DNA methyltransferases, we detected the main effect of treatment on luteal expression of DNMT1 gene and protein, DNMT3A protein, and DNMT3B gene and protein (E2-treated groups vs. the control group, *p* < 0.05). No main effect of the site of hormone administration (i.e., the placebo-treated uterine horn vs. the hormone-treated uterine horn within the same animal) on luteal gene expression was observed (*p* > 0.05). Luteal tissues collected from gilts on day 12 of the estrous cycle and pregnancy were used as referential groups. E2 administered into the uterine lumen (833 ng/infusion) increased (*p* < 0.05) luteal expression of the *DNMT1* gene regardless if CL was adjacent or contralateral to E2-infused uterine horn compared to CLs collected from the control group receiving placebo (Figure 2A). An intrauterine infusion of E2 (33.3 µg/infusion) decreased the luteal expression of the *DNMT3B* gene compared to the control group (*p* < 0.05; Figure 2E). Likewise, the luteal expression of the *DNMT3B* gene was decreased in CLs collected from gilts on day 12 of pregnancy compared to gilts on day 12 of the estrous cycle (*p* < 0.05; Figure 2E). No significant differences in the luteal expression of the *DNMT1* and *DNMT3A* genes were observed in samples collected from gilts on day 12 of pregnancy and the estrous cycle (*p* > 0.05; Figure 2A,B).

Elevated expression of the DNMT1 protein was found in CLs collected from gilts receiving E2 (33.3 µg/infusion) compared to gilts from the control group (*p* < 0.05; Figure 2B). Estradiol-17β (833 ng/infusion) lowered the luteal expression of the DNMT3A and DNMT3B proteins compared to gilts receiving placebo infusions into both uterine horns (Figure 2D,F). Similarly, decreased expression of luteal DNMT3A and DNMT3B proteins was observed in CLs collected from gilts on day 12 of the pregnancy compared to gilts on day 12 of the estrous cycle (*p* < 0.05; Figure 2D,F). No significant difference in luteal expression of the DNMT1 protein was observed in samples collected from gilts on day 12 of pregnancy and the estrous cycle (*p* > 0.05; Figure 2B).

### 2.3. Estradiol-17β Alters Expression of Genes Involved in Luteal Function In Vivo

**Figure 3:**
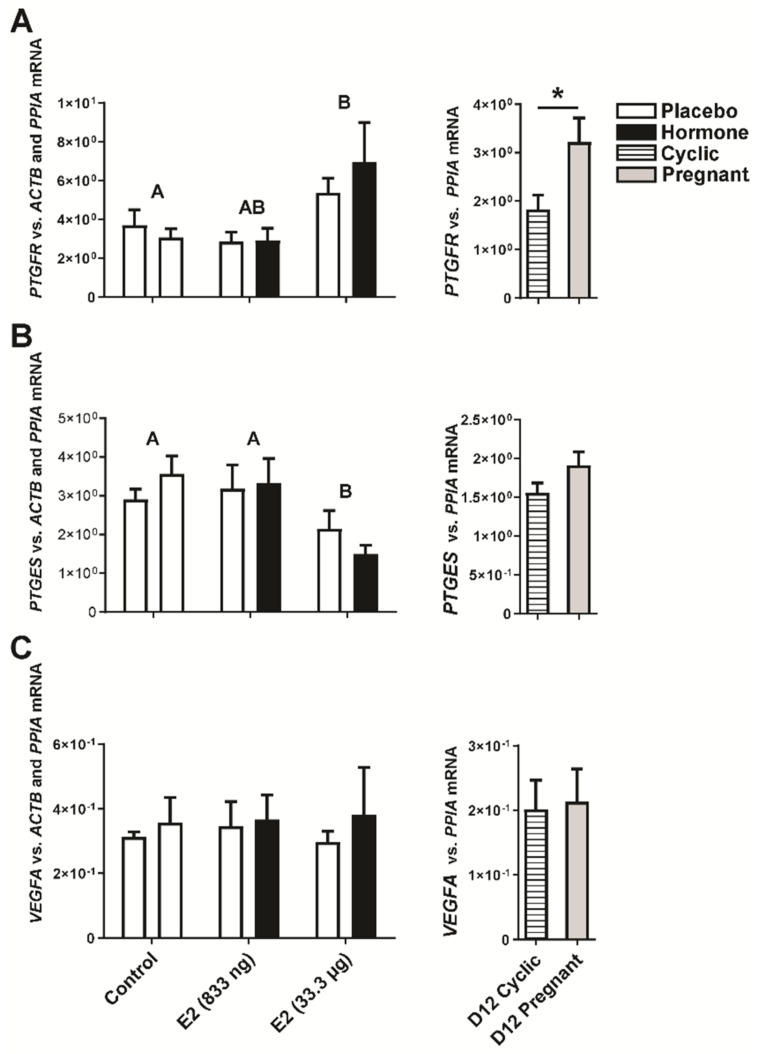
Expression of prostaglandin F2α receptor (*PTGFR*; (**A**)), prostaglandin E2 synthase (*PTGES*; (**B**)) and vascular endothelial growth factor (*VEGFA*; (**C**)) genes in the corpora lutea collected from estradiol-17β (E2)- and placebo-treated gilts. Control—gilts receiving placebo infusions into both uterine horns. E2—gilts receiving infusions of either placebo into one randomly selected horn or E2 (833 ng or 33.3 µg/infusion) into the contralateral horn. As referential groups, gilts on day 12 of the estrous cycle (D12 Cyclic) and pregnancy (D12 Pregnant) were included in analyses. Values of target gene expression were normalized against geometrical mean of *ACTB* and *PPIA* expression values (luteal samples from in vivo model) or *PPIA* expression values (luteal samples from day 12 pregnant/cyclic pigs). Main effect of treatment was detected on luteal expression of *PTGFR* and *PTGES* mRNA (E2-treated groups vs. the control group; *p* < 0.05). There was no significant effect of the site of hormone administration (i.e., the placebo-treated uterine horn vs. the hormone-treated uterine horn within the same animal) on luteal expression of *PTGFR*, *PTGES,* and *VEGFA* mRNA. Data are expressed as the mean ± SEM. Different letters indicate statistically significant differences between control- and hormone-treated gilts (*p* < 0.05). Asterisk indicate statistical differences between gilts on day 12 of the estrous cycle and pregnancy (*p* < 0.05).

Evaluating quantitative PCR analysis results we detected the main effect of E2 treatment on luteal expression of *PTGFR*, *PTGES*, *STAR*, *HSD17B1*, *CYP19A1,* and *PGRMC1* genes (E2-treated groups vs. the control group, *p* < 0.05). However, there was no significant effect of the site of hormone administration (i.e., the placebo-treated uterine horn vs. the hormone-treated uterine horn within the same animal) on luteal gene expression (*p* > 0.05). Intrauterine administration of E2 affected the expression of genes vital for proper functioning of the corpus luteum. We found that E2 (33.3 µg of E2/ infusion) increased luteal expression of *PTGFR* (*p* < 0.05; Figure 3A) regardless if CL was adjacent or contralateral to E2-infused uterine horn. Accordingly, increased levels of *PTGFR* mRNA were observed in CLs collected from gilts on day 12 of pregnancy compared to day 12 of the estrous cycle (*p* < 0.05, Figure 3A). Decreased expression of PGE2 synthase (*PTGES*; *p* < 0.05, Figure 3B) was observed in CLs collected from ovaries adjacent and contralateral to the uterine horn treated with E2 (33.3 µg of E2/ infusion) compared to CLs collected from gilts receiving placebo infusions into both uterine horns (the control group; Figure 3B). The luteal expression of *VEGFA* was unaffected both in CLs collected from gilts on day 12 of the estrous cycle/pregnancy, and in CLs collected from E2- and placebo-treated gilts (*p* > 0.05; Figure 3C).

**Figure 4:**
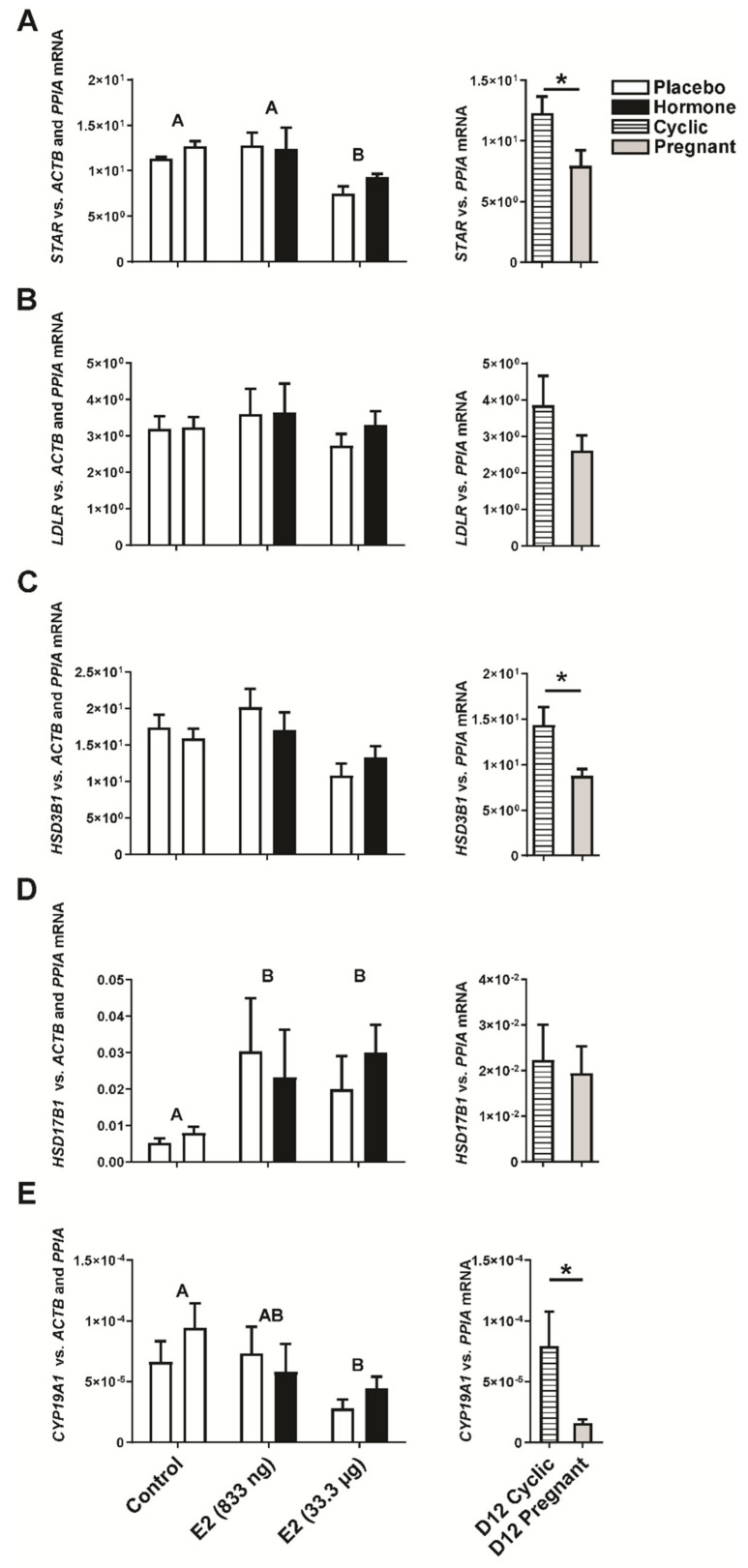
Expression of steroidogenic acute regulatory protein (*STAR*; (**A**)), low density lipoprotein receptor *(LDLR*; (**B**)), hydroxy-delta-5-steroid dehydrogenase, 3 beta- and steroid delta-isomerase 1 (*HSD3B1*; (**C**)), hydroxysteroid 17-beta dehydrogenase (*HSD17B1*; (**D**)) and cytochrome P450 family 19 subfamily A member 1 (*CYP19A1*; (**E**)) genes in the corpora lutea collected from estradiol-17β (E2)- and placebo-treated gilts. Control—gilts receiving placebo infusions into both uterine horns. E2—gilts receiving infusions of either placebo into one randomly selected horn or E2 (833 ng or 33.3 µg/infusion) into the contralateral horn. As referential groups, gilts on day 12 of the estrous cycle (D12 Cyclic) and pregnancy (D12 Pregnant) were included in analyses. Values of target gene expression were normalized against geometrical mean of *ACTB* and *PPIA* expression values (luteal samples from in vivo model) or *PPIA* expression values (luteal samples from day 12 pregnant/cyclic pigs). Main effect of treatment was detected on luteal expression of *STAR*, *HSD17B1,* and *CYP19A1* mRNA (E2-treated groups vs. the control group; *p* < 0.05). There was no significant effect of the site of hormone administration (i.e., the placebo-treated uterine horn vs. the hormone-treated uterine horn within the same animal) on luteal expression of analyzed genes. Data are expressed as the mean ± SEM. Different letters indicate statistically significant differences between control- and hormone-treated gilts (*p* < 0.05). Asterisk indicates statistical differences between gilts on day 12 of the estrous cycle and pregnancy (*p* < 0.05).

The luteal expression of the *STAR* and *CYP19A1* genes was found to be down-regulated by the intrauterine infusion of E2 (33.3 µg of E2/ infusion; *p* < 0.05; Figure 4A,E) regardless if CL was adjacent or contralateral to E2-infused uterine horn. In CLs collected from gilts on day 12 of pregnancy, the expression of *STAR, HSD3B1*, *CYP19A1* was lower compared to CLs collected from gilts on day 12 of the estrus cycle (*p* < 0.05; Figure 4A,C,E). We found a stimulating effect of E2 infused into the uterine lumen (33.3 µg of E2/infusion) on the luteal expression of the hydroxysteroid 17-beta dehydrogenase 1 (*HSD17B1*) gene (*p* < 0.05; Figure 4D), however, its expression remained unchanged in CLs collected from gilts on day 12 of pregnancy or the estrous cycle (*p* > 0.05; Figure 4D). There was no difference in low-density lipoprotein receptor (*LDLR*) gene expression in CLs collected from E2- and placebo-treated gilts and gilts on day 12 of pregnancy or the estrous cycle (Figure 4B).

**Figure 5:**
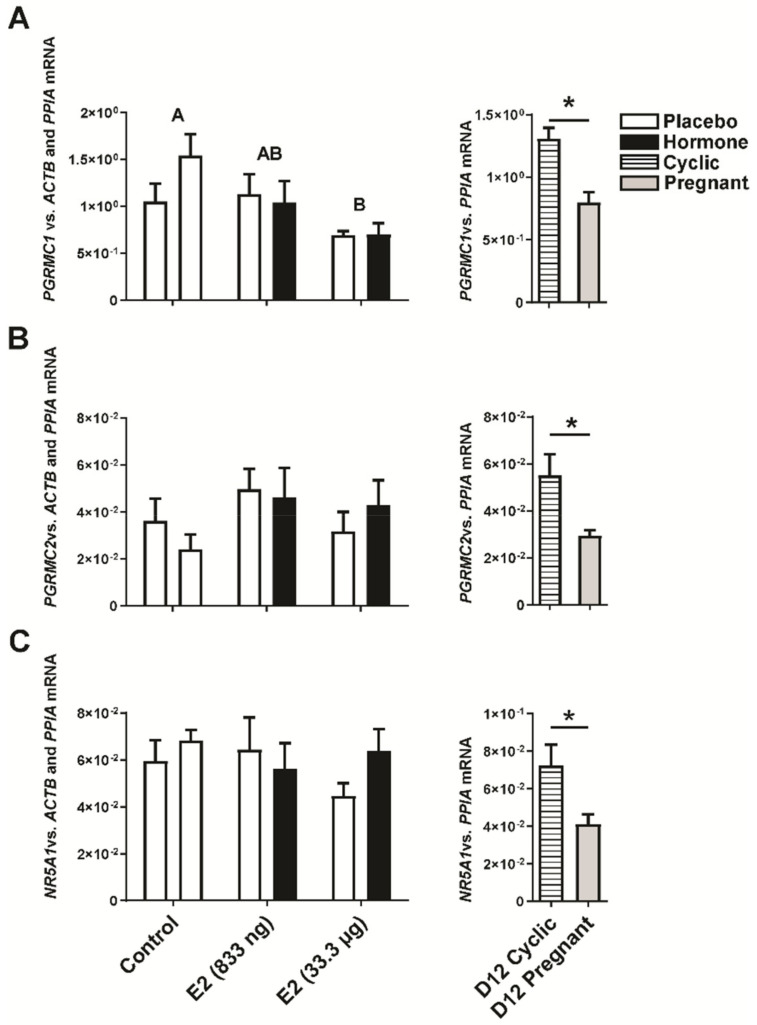
Expression of progesterone receptor membrane component 1 (*PGRMC1*; (**A**)), and 2 (*PGRMC2*; (**B**)), and nuclear receptor subfamily 5 group A member 1 (*NR5A1*; (**C**)) genes in the corpora lutea collected from E2- and placebo-treated gilts. Control—gilts receiving placebo infusions into both uterine horns. E2—gilts receiving infusions of either placebo into one randomly selected horn or E2 (833 ng or 33.3 µg/infusion) into contralateral horn. As referential groups, gilts on day 12 of the estrous cycle (D12 Cyclic) and pregnancy (D12 Pregnant) were included. Values of target gene expression were normalized against geometrical mean of *ACTB* and *PPIA* expression values (luteal samples from in vivo model) or *PPIA* expression values (luteal samples from day 12 pregnant/cyclic pigs). Main effect of treatment was detected on luteal expression of *PGRMC1* mRNA (E2-treated groups vs. the control group; *p* < 0.05). There was no significant effect of the site of hormone administration (i.e., the placebo-treated uterine horn vs. the hormone-treated uterine horn within the same animal) on luteal expression of analyzed genes. Data are expressed as the mean ± SEM. Different letters indicate statistically significant differences between control- and hormone-treated gilts (*p* < 0.05). Asterisk indicates statistical differences between gilts on day 12 of the estrous cycle and pregnancy (*p* < 0.05).

Estradiol-17β infused into the uterine lumen lowered the luteal expression of *PGRMC1* (*p* < 0.05; Figure 5A) but not *PGRMC2* (*p* > 0.05; Figure 5B) nor *NR5A1* (*p* < 0.05; Figure 5C), whereas decreased levels of either *PGRMC1*, *PGRMC2*, or *NR5A1* transcripts were observed in CLs collected from gilts on day 12 of the pregnancy compared to day 12 of the estrous cycle (*p* < 0.05; Figure 5A–C).

## 3. Discussion

The present report is the first evidencing the in vivo effect of the primary conceptus signal (estradiol-17β) on the luteal expression of genes linked to processes pivotal for corpus luteum functioning in the pig. Moreover, we indicated a new perspective for E2 to regulate the processes related to DNA methylation as possible physiological mechanisms controlling the expression of genes in porcine CLs.

Embryonic estrogens in the pig have been implied to play a luteoprotective role. Indeed, by acting on CL, E2 stimulates progesterone secretion [9]. Exogenous administration of E2 resulted in maintenance of a high expression of luteal LH receptors [8]. The corpus luteum is the key endocrine organ determining successful establishment of pregnancy in mammals. Its main function is synthesis and secretion of progesterone, which is responsible for priming uterine receptivity. Intriguingly, results from studies involving *CYP19A1*-deficient porcine embryos have contested the role of E2 in pregnancy establishment until day 30–35. However, our studies using the in vivo model of intrauterine E2 administration revealed that E2 was responsible for substantial changes within the endometrial transcriptome, affecting the processes essential for conceptuses development and implantation [24]. The importance of E2 as a factor involved in the maintenance of CL function is also supported by previous studies involving in vivo models of systemic E2 administration and its influence on CL function, resulting in so-called pseudopregnancy in pigs [8,9,25,26]. Herein, using the novel in vivo model which better reflects the physiological levels of the conceptus signal (E2) and route of its administration, we showed that an intrauterine infusion of E2 resulted in elevated levels of E2 in the luteal tissue that, in turn, suggests that E2 is transferred from the uterine lumen through local circulation into the corpora lutea. It is worth noting that elevated levels of E2 in the CL (approximately 11.17–20 pg/mg of luteal tissue) were similar to those observed in CLs collected from gilts on day 12 of the pregnancy (approximately 17.5 pg/mg of luteal tissue; [15]). Furthermore, increased levels of progesterone were found in CLs collected from pigs receiving intrauterine infusions of E2 compared to gilts from the control group receiving placebo (vehicle) infusions. These results are in line with previous reports indicating increased luteal concentration of P4 in porcine CLs on day 12 compared to earlier stages of pregnancy [27]. Moreover, our results confirm previous statements that E2 stimulates luteal P4 secretion [28]. Thus, it can be stated that our in vivo model of intrauterine infusions of E2 accurately reflects the signalization of conceptuses during the time corresponding to the maternal recognition of pregnancy.

Estradiol-17β can exert its function by the genomic pathway involving nuclear receptors ESR1 and ESR2 [29,30], or the non-genomic pathway involving G protein-coupled receptors (GPER1, GPR30) [31,32]. However, genomic and non-genomic effects of E2 are not the only mechanism by which E2 may regulate the expression of particular genes. Studies on tumor growth have demonstrated the great potential of E2 in the induction of changes involving DNMT1, DNMT3A, and DNMT3B related to DNA methylation [16,17]. DNMT1 is responsible for the maintenance of inherited DNA methylation patterns, while DNMT3A and DNMT3B maintain and correct the errors of DNMT1 [17], but also are involved in de novo DNA methylation [33]. Similar to cancer development, the growth of luteal tissue is highly dynamic. Hence, we hypothesized that the E2-induced epigenetic mechanisms involved in the development of tumors [18,19] may also regulate physiological processes occurring in the functioning corpus luteum. In the present study, we showed for the first time, in any species, that intrauterine infusion of E2 resulted in down-regulation of the DNMT3A and DNMT3B protein in porcine CLs in vivo. A similar pattern was also observed in CLs collected on day 12 of pregnancy when compared to CLs collected on day 12 of the estrous cycle. On the other hand, intrauterine administration of E2 led to stimulated expression of the DNMT1 gene and protein. These results suggest that E2, by differential regulation of DNMTs in the porcine CL, may be responsible for maintenance of inherited DNA methylation patterns. However, this hypothesis requires further study. According to our knowledge, there are scant reports concerning changes in DNA methylation in the CL. The present results are consistent with previous findings indicating that the expression of some factors involved in ovarian function, such as *CYP19* [20,21], oxytocin [22], or inhibin α [23], has been altered due to methylation of DNA sequences.

In the present study, we elucidated the in vivo effect of E2 on the expression of genes involved in the function of the porcine corpora lutea. Endometrial prostaglandin F2α (PGF2α) and E2 (PGE2) exert opposing actions on the corpus luteum after the acquisition of luteolytic sensitivity. Therefore, the proper control over their synthesis, secretion, and metabolism is essential either for the initiation of luteolysis or for the maintenance of pregnancy in mammalian species, including pigs (reviewed in [3,14]). In pigs, the corpus luteum is protected from luteolysis through the synthesis of large amounts of luteoprotective PGE2 by conceptuses and the endometrium prior to implantation [34,35]. Surprisingly, recent studies have postulated that PGF2α might also be involved in luteal function maintenance during early pregnancy in pigs, by stimulation of angiogenesis [15]. Elevated expression of PTGFR mRNA and protein has been observed in CLs on day 12 of pregnancy compared to day 12 of the estrous cycle [15]. A stimulating effect of PGF2α on luteal angiogenesis has been described in bovines [36,37]. Our previous studies revealed that E2 regulates the expression of the endometrial PTGFR gene and protein in the pig, and indicated the involvement of PGF2α-PTGFR signaling in angiogenic changes within the endometrium [11,12,38]. Likewise, in the present study, we found a stimulating effect of an intrauterine infusion of E2 on luteal *PTGFR* expression in vivo. This result supports the previous hypothesis that increased expression of PTGFR may be linked with the role of PGF2α-PTGFR signaling in luteal angiogenesis. Interestingly, the mechanisms by which E2 regulates the expression of PTGFR are still unclear. Based on the candidate sequences for estrogen-responsive elements (ERE) [39], we searched the DNA sequence of PTGFR and no such sequences were identified. However, results from methylome profiling of CLs revealed several hypomethylated regions within the *PTGFR* DNA sequence, localized in the promoter region or in intron 1 in CLs collected during the estrus phase, compared to those collected during the proestrus phase [40]. Hence, it could be speculated that estrogen-affected upregulation of the *PTGFR* gene on day 12 of pregnancy may be due to differential methylation of its DNA sequence in response to high luteal E2 secretion by conceptus.

Our results indicate that intrauterine infusion of E2 resulted in down-regulated expression of the PGE2 synthase gene (*PTGES*). These results are in line with those of a previous study reporting decreased levels of *PTGES* mRNA in CLs collected on day 12 of pregnancy, compared to day 12 of the estrous cycle [15]. These results also confirm the previous hypothesis assuming that synthesis of PGE2 in the porcine conceptus and endometrium, rather than in the corpus luteum, may be involved in the rescue of the corpus luteum during maternal recognition of pregnancy [41]. Thus, it has been indicated that PGE2 of conceptus and the endometrium origin is transferred via local blood and lymph circulation to the ovaries [42], where it may exert its luteoprotective effect [43,44] through PTGER2 and PTGER4 [13,15]. To the best of our knowledge, there are no reports elucidating the molecular mechanisms by which E2 may decrease the expression of *PTGES*. Results from luteal methylome profiling revealed a hypermethylated sequence localized in the exon 3 of the porcine *PTGES* DNA sequence in CLs collected during the estrus phase compared to the proestrus phase [40]. Thus, we presume that E2-affected down-regulation of luteal *PTGES* expression in pigs may be related to hyper-methylation of its DNA sequence.

The steroidogenic acute regulatory protein (STAR) is a transport protein that regulates cholesterol transfer within the mitochondria, which is the rate-limiting step in the production of steroid hormones [45]. In the present report, we noted a decreased luteal expression of the *STAR* gene in response to intrauterine E2 treatment. Similarly, lower levels of *STAR* mRNA were observed in CLs collected from gilts on day 12 of the pregnancy compared to CLs collected on day 12 of the estrous cycle. Interestingly, differential methylation of the *STAR* DNA sequence has been reported in caprine ovaries. The methylation ratio of the *STAR* gene was lower in the estrus phase compared to the diestrus phase [46]. On the other hand, no differences in luteal *STAR* expression on day 12 of the estrous cycle and pregnancy have been observed in previous reports [15]. However, a significant increase in luteal *STAR* expression has been noted in later days of the pregnancy (day 14) [15]. Thus, several mechanisms potentially regulating the luteal STAR expression are possible. The first is that the expression of *STAR* is controlled by E2-induced epigenetic regulation; the second is the existence of a time-shift occurring between secretion of E2 and its effect; and the third is that the expression of STAR is controlled by factors other than E2. Therefore, extended studies on mechanisms by which the luteal expression of STAR is controlled are required.

Hydroxysteroid 17-beta dehydrogenase 1 is an enzyme responsible for the interconversion of estrone (E1) and estradiol (E2). It can use E1 as substrate from both the aromatase and sulfatase pathways, and it synthesizes E2 using reduced nicotinamide adenine dinucleotide as a cofactor [47]. Our results indicate elevated expression of *HSD17B1* in the porcine CLs in response to E2 treatment. Given the fact that both the expression and activity of HSD17B1 are significantly higher in the growth of breast tumors [48], it can be speculated that E2 may support dynamic growth of the CLs during early pregnancy. On the other hand, we found no differences in the expression of *HSD17B1* in the CLs collected on day 12 of pregnancy and the estrous cycle. Similar results were demonstrated by Przygrodzka and coworkers [15]. The DNA sequence corresponding to the *HSD17B1* gene was found to be hypomethylated in the porcine ovaries during the proestrus phase (when E2 levels are higher) compared to the estrus phase [40]. Thus, it is possible that E2-stimulated expression of *HSD17B1* in the porcine CLs may also be related to differential methylation of its DNA sequence induced by E2. This hypothesis, however, needs further study.

Aromatase is a key enzyme involved in the conversion of androgens into estrogens. Herein, we observed a down-regulation of the *CYP19A1* gene in the CLs collected from animals receiving intrauterine infusions of E2, and accordingly on day 12 of pregnancy. These results may indicate the existence of a negative autoamplification loop by which E2 controls its own synthesis in the porcine CLs during early pregnancy. Interestingly, increased methylation of *CYP19A1* promoter has been shown in bovine CLs during the early luteal phase [21] and at month 5 of pregnancy [20]. Hence, the question arises whether an E2-affected decrease in *CYP19A1* expression in the porcine CL may be related to the change of its DNA sequence methylation.

Progesterone mediates its actions via nuclear and membrane receptors (PGR and PGRMC1, PGRMC2, respectively). The expression of PGRMC1 has been indicated to be essential to enhance steroidogenesis [49] and to sustain antiapoptotic mechanisms mediated by P4 in luteal cells [50]. Our results indicate decreased expression of *PGRMC1* in the CLs collected from gilts receiving E2 infusions compared to control gilts receiving infusions of a placebo. No effect of E2 has been observed on luteal *PGRMC2* expression in vivo. Interestingly, lowered expression of *PGRMC1* and *PGRMC2* has been found in CLs on day 12 of pregnancy compared to day 12 of the estrous cycle, which is in line with previously reported results for *PGRMC2* gene expression on day 12 of pregnancy [15]. On the other hand, increased expression of *PGRMC1* on day 14 of pregnancy [15] may suggest the existence of a time shift necessary for E2-affected regulation of *PGRMC1* gene expression.

In conclusion, the present report suggests the primary conceptus signal (estradiol-17β) transferred from the uterine lumen into the ovaries affects the luteal expression of genes related to processes vital for corpus luteum function in vivo in the pig. Moreover, we indicated for the first time a new potential for E2 to regulate the processes related to DNA methylation as putative physiological mechanisms controlling the luteal expression of genes, that so far have not been described in any species.

## 4. Materials and Methods

All procedures involving the use of animals were conducted in accordance with the national guidelines for agricultural animal care and were approved by the Animal Ethics Committee, University of Warmia and Mazury in Olsztyn, Poland, permission No. 17/2008.

### 4.1. Tissue Collection

#### 4.1.1. Animal Model In Vivo

To determine the effect of intrauterine infusions of E2 on porcine corpora lutea we used in vivo model described in our previous studies [12,24,51]. Prepubertal crossbred gilts (Pietrain × Duroc) of similar age (6 months) and genetic background from the same herd were observed for the onset of the estrous cycle. Following one natural estrous cycle, after the second natural estrus, gilts were subjected to the synchronization protocol in which gilts received intramuscular between days 12-14 of the estrous cycle gilts were injected with 10 mg PGF2α (Dinolytic; Pfizer, Warsaw, Poland). Sixteen hours later, 10 mg of PGF2α was injected simultaneously with 750 IU PMSG (Folligon; Intervet, Boxmeer, The Netherlands). After 72 h, 500 IU hCG (Chorulon; Intervet) was given intramuscularly. On day 8–9 of the third estrous cycle animals underwent surgery as described previously [12]. Hormone delivery by conceptuses was simulated by perforated cannulas placed surgically into the uterine lumen at a distance of 10–15 cm from the isthmus. Following surgery, animals were divided into three groups. Animals from the control group (*n* = 7), received placebo (5 mL of 1% *v*/*v* ethanol saline) infusions into each uterine horn. The doses of hormones used in the experimental groups were similar to those previously published [26,28]. Gilts from the experimental groups received hormonal infusions: 833 ng of E2 per infusion (*n* = 6) or 33.3 µg of E2 per infusion (*n* = 5) to randomly selected uterine horn whereas the contralateral horn received infusions of placebo. Administration of treatments was performed every 4 h for 24 h on days 11–12 after the onset of estrus. Following the experiment, gilts were slaughtered at the local abattoir. Ovaries adjacent to placebo- and hormone-treated horns were dissected by scissors. Randomly selected corpora lutea were separated from each ovary. Luteal tissue was dissected from surrounding ovarian tissue and collected from the central part of CL and snap-frozen in liquid nitrogen. Collected samples were stored in −80°C for further analyses. Any inflammatory changes detected in collected uteri and/or intrauterine fluid accumulation excluded the material from further procedures. The effect of E2 on DNA-methyltransferase 1, 3A, and 3B (DNMT1, DNMT3A, and DNMT3B, respectively) protein expression was studied by Western blot as described in Section 4.3. The effects of E2 on *DNMT1*, *DNMT3A*, *DNMT3B,* and genes involved in CL function (*PTGFR*, *PTGES*, *VEGFA*, *STAR*, *LDLR*, *HSD3B1*, *HSD17B1*, *CYP19A1*, *PGRMC1*, *PGRMC2,* and *NR5A1*) were studied using quantitative polymerase chain reaction (real-time RT PCR; qPCR) as described Section 4.4.

#### 4.1.2. Animal Model Ex Vivo

Gilts on day 12 of the estrous cycle and pregnancy were used as the referential groups. First, prepubertal crossbred gilts were observed for the onset of the estrous cycle. Following two naturally-occurred estrous cycles, gilts were divided into two groups: cyclic and pregnant. Gilts assigned to “pregnant” group were mated twice 24 h and 48 h after onset of estrus. Gilts on day 12 of the estrous cycle (*n* = 7) and pregnancy (*n* = 7) were slaughtered in the local abattoir. Pregnancy was confirmed by the presence of conceptuses. The conceptuses were flushed from each uterine horn with sterile phosphate-buffered saline. Luteal tissue was separated as described in Section 4.1.1. and snap-frozen in liquid nitrogen and stored in −80 °C for further analyses. Expression of DNMT1, DNMT3A, and DNMT3B protein was determined using Western blot as described in Section 4.3. The effects of E2 on *DNMT1*, *DNMT3A*, *DNMT3B,* and genes involved in CL function (*PTGFR*, *PTGES*, *VEGFA*, *STAR*, *LDLR*, *HSD3B1*, *HSD17B1*, *CYP19A1*, *PGRMC1*, *PGRMC2,* and *NR5A1*) were studied using qPCR as described in Section 4.4.

### 4.2. The Effect of E2 on Luteal Estradiol-17β and Progesterone Abundance

Levels of E2 and P4 in luteal tissue in response to intrauterine treatment with E2 were determined by radioimmunoassay as described previously [15,52]. Briefly, luteal tissues were homogenized in 5% trichloroacetic acid (1 mL of 5% TCA per 1 mg of luteal tissue) using IKA Ultra-Turrax homogenizer (IKA^®^-Werke GmbH & Co. KG; Staufen, Germany). Homogenates were then centrifuged (10,000× *g*, 10 min, 4 °C) and supernatants were collected and neutralized with NaOH (2.5 mM) to pH 7.5. The abundance of E2 and P4 was then determined using radioimmunoassay kits (DIAsource, Louvain-la-Neuve, Belgium) accordingly to the manufacturer’s protocol. The sensitivity of the E2 and P4 assays were 3 pg/mL, and 0.05 ng/mL, respectively. The intra-assay coefficient of variations (CV) for the E2 and P4 assays were 6.8% and 5.5%, respectively.

### 4.3. Western Blot Analyses

The expression of DNMT1, DNMT3A, and DNMT3B proteins in luteal tissue collected from gilts after in vivo experiment and gilts on day 12 of the estrous cycle and pregnancy was determined using Western blot. Nuclear extracts from luteal tissue fragments (approx. 300 mg) were prepared using Nuclear Extraction Kit (Cayman Chemical, Ann Arbor, MI, USA) according to the manufacturer’s protocol. Nuclear extracts (40 µg) were dissolved in SDS gel-loading buffer (50 mM/L Tris–HCl, pH 6.8; 4% SDS, 20% glycerol, and 2% β-mercaptoethanol) and denatured at 95 °C for 4 min. Samples were loaded onto 4–20% stain-free gels (Bio-Rad Laboratories, Hercules, CA, USA) and separated by SDS PAGE. Separated proteins were then electrotransfered onto 0.2 mm PVDF membrane (Millipore, Burlington, MA, USA) in transfer buffer (20 mM/L Tris–HCl buffer, pH 8.2; 150 mM/L glycine, and 20% methanol). Membranes were blocked in 5% non-fat dry milk in Tris-buffered saline buffer (TBS-T, containing 0.1% Tween-20) for 90 min at 25.6 °C. Subsequently, the membranes were incubated overnight with primary antibodies (Table 1).

Following incubation, membranes were washed three times in TBST-T and afterwards incubated with secondary antibodies (Table 1) dissolved 1:20,000 in TBS-T, at room temperature for 90 min. For anti-DNMT1 antibodies secondary anti-mouse antibodies (Table 1) were used. For DNMT3a and DNMT3B antibodies, secondary anti-rabbit antibodies (Table 1) were applied. After incubation membranes were washed three times in fresh TBS-T. Immune complexes were visualized using Clarity Western ECL Substrate (Bio-Rad) and archived by ChemiDoc Imaging System (Bio-Rad). To calculate DMNT protein expression, values of chemiluminescence signals reflecting DNMT1, DNMT3A, and DNMT3B protein expression levels were divided by values of total protein measurement using Image Lab 6.0 (Bio-Rad) algorithm.

### 4.4. Quantitative PCR Analyses

#### 4.4.1. Total RNA Isolation and cDNA Synthesis

Total RNA was isolated from luteal tissue fragments (approx. 50 mg) collected from gilts after in vivo experiment and from gilts on day 12 of the estrous cycle and pregnancy and reverse-transcribed as described previously [11]. Concentration and integrity of isolated RNA was determined by Agilent 2100 Bioanalyzer (Agilent Technologies, Santa Clara, CA, USA) and NanoDrop spectrophotometer (Thermo Fisher Scientific; Waltham, MA, USA). The RNA Integrity Numbers (RIN) of isolated RNA samples were above 8.9. Isolated RNA was reverse-transcribed by using MultiScribe™ Reverse Transcriptase (Thermo Fisher Scientific). Synthesized cDNA samples were stored in −80 °C for further real-time RT-PCR analyses.

#### 4.4.2. Real-Time RT-PCR

Analysis of gene expression in luteal tissues was performed as described previously [11,12]. Expression of *DNMT1*, *DNMT3A* and *DNMT3B*, *ACTB*, *GAPDH,* and *PPIA* genes expression was quantified using Power SYBR green (Thermo Fisher Scientific) with specific primers (1 µM; Table 2) whereas *PTGFR*, *PTGES*, *VEGFA*, *STAR*, *LDLR*, *HSD3B1*, *HSD17B1*, *CYP19A1*, *PGRMC1*, *PGRMC2,* and *NR5A1* genes were analyzed using TaqMan^®^ mastermix with TaqMan probes (Thermo Fisher Scientific; Table 2) according to the manufacturer’s protocol.

Following PCR program for *DNMT1*, *DNMT3A*, *DNMT3B* genes and for genes analyzed using TaqMan assays was performed: initial denaturation (95 °C, 10 min) followed by 40 cycles of denaturation (95 °C; 15 s), annealing and elongation (60 °C; 1 min). For *ACTB*, *PPIA* and *GAPDH* examined using SYBR Green the following PCR program was applied: initial denaturation (95 °C, 10 min) followed by 36 cycles of denaturation (95 °C, 15 s), annealing (55 °C, 30 s) and elongation (72 °C, 1 min). For genes amplified using SYBR Green, after PCR, melting curve analysis was done to ensure that a single product was amplified in the PCR reaction. All real-time PCR reactions were performed by using Applied Biosystems 7900HT Real-Time PCR system (Life Technologies; Carlsbad, CA, USA). Gene expression analysis was performed by relative qPCR quantification method described by Zhao and Fernald [53] and by using real-time PCR Miner software [53]. The stability of the reference genes in the luteal samples was evaluated using the statistical algorithm NormFinder 2.0 [54]. Analyses were done separately for tissues collected from the in vivo model and for luteal samples collected from gilts on day 12 of the estrous cycle and pregnancy. Stability of three reference genes: *ACTB*, *GAPDH*, and *PPIA* was analyzed. Gene expression values in luteal tissues collected from gilts after in vivo experiment were calculated by dividing target gene expression value by the geometrical mean of *PPIA* and *ACTB* expression values. Gene expression values in luteal tissues collected from gilts on day 12 of pregnancy and the estrous cycle were calculated by dividing the expression value of the target gene by the expression values of *PPIA* gene.

**Table 2:** Primer sequences and TaqMan^®^ assays used in real-time RT-PCR analyses.

Gene	Primer Sequence/TaqMan^®^ Assay ID	Reference
*DNMT1*	Sense: 5′-TGGCGGGACCTACCAAACA-3′ Antisense: 5′-ACTTCCACGCAGGAGCAGA-3′	[55]
*DNMT2*	Sense: 5′-AAGAATGCCACCAAATCAGCC-3′ Antisense: 5′-AGAACTTGCCGTCTCCGAACCA-3′	
*DNMT3*	Sense: 5′-AGGTCTCCAGCCTCCTAAGTT-3′ Antisense: 5′-GTGTCTGAGCCATCTCCATCC-3′	
*ACTB*	Sense: 5′-ACATCAAGGAGAAGCTCTGCTACG-3′ Antisense: 5′-GAGGGGCGATGATCTTGATCTTCA-3′	[11]
*GAPDH*	Sense: 5′-CAGCAATGCCTCCTGTACCA-3′ Antisense: 5-GATGCCGAAGTTGTCATGGA-3′	
*PPIA*	Sense: 5′-TAACCCCACCGTCTTCTT-3′ Antisense: 5′-TGCCATCCAACCACTCAG-3′	
*PTGFR*	Ss03393819_s1	[15]
*PTGES*	Ss03392129_m1	
*VEGFA*	Ss03393993_m1	
*STAR*	Ss03381250_u1	
*LDLR*	Ss03373254_u1	
*HSD3B1*	Ss03391752_m1	
*HSD17B1*	Ss04245960_g1	
*CYP19A1*	Ss03384876_u1	
*PGRMC1*	Ss03392695_u1	
*PGRMC2*	Ss03388999_m1	
*NR5A1*	Ss03394295_m1	
*ACTB*	Ss03376081_u1	
*GAPDH*	Ss03373286_u1	
*PPIA*	Ss03394782_g1	

### 4.5. Statistical Analyses

Results of hormones (E2 and P4) abundance, as well as gene and protein expression in luteal samples collected from gilts after in vivo experiments, were analyzed using Two-way ANOVA, followed by Bonferroni post-test. Main effects: the effect of treatment between groups (placebo-treated vs. 833 ng E2-treated vs. 33.3 µg E2-treated) and the effect of the site of hormone administration (i.e., corpora lutea adjacent to the placebo-treated uterine horn vs. corpora lutea adjacent to the hormone-treated uterine horn) on luteal hormone concentration as well as on gene and protein expression were assessed. Because no main effect of the site of hormone administration was detected, we analyzed the effect of treatment by Bonferroni post-test (control gilts vs. low dose E2-treated gilts vs. high dose E2-treated gilts). Gene and protein expression in luteal samples collected from gilts on day 12 of the estrous cycle and pregnancy was analyzed using t-test. All data were tested for normality and homoscedasticity. Log-transformation of data was applied before performing parametric tests when needed. Differences were considered as statistically significant at the 95% confidence level (*p* < 0.05). All statistical analyses were conducted using GraphPad PRISM v. 9.0.0. software (GraphPad Software Inc., San Diego, CA, USA).

## Figures and Tables

**Table 1:** List of antibodies used in Western blot analyses.

Peptide/Protein Target	Antigen Sequence	Name of Antibody	Manufacturer, Catalog No., or Name of Source	Species Raised in Monoclonal or Polyclonal	Dilution Used
DNMT1	EKDDREDKENAFKR	DNMT1 Antibody	NB100-56519 Novus Biologicals	Mouse, monoclonal	1:1000
DNMT3A	N/A	DNMT3A (D23G1) Rabbit mAb	#3598 Cell Signaling Technology	Rabbit, monoclonal	1:300
DNMT3B	RGRRSSSRLSKREVSSC	DNMT3B Antibody	orb372330 Biorbyt	Rabbit, polyclonal	1:100
Anti-mouse, secondary antibodies	N/A	Immun-Star Goat Anti-Mouse (GAM)-HRP Conjugate	Bio-Rad; 1705047	Goat, polyclonal	1:20 000
Anti-rabbit, secondary antibodies	N/A	Immun-Star Goat Anti-Rabbit (GAM)-HRP Conjugate	Bio-Rad; 1706515	Goat, poyclonal	1:20 000

## Data Availability

The data presented in this study are available on request from the corresponding authors.
